# Supplementary material for: In-utero exposure to chikungunya and child morbimortality: a population-based study using linked routine data
Source: Nat Commun. 2026 Mar 21;17:4581. doi: 10.1038/s41467-026-70786-5 (PMC13194738; doi:10.1038/s41467-026-70786-5)
Supplement: Supplementary file 1 — Supplementary Information [file 41467_2026_70786_MOESM1_ESM.pdf]

Supplementary Materials

In-Utero Exposure to Chikungunya and Child Morbimortality: A Population-Based Study Using Linked Routine Data

Authors:

Mio Kushibuchi*[1]; Orlagh Carroll[2]; Thiago Cerqueira-Silva[2,3]; Viviane S. Boaventura[3, 4]; Maria Glória Teixeira[5]; Mauricio L Barreto[1]; and Enny S Paixão[1, 2]

Affiliations:

1 Centre for Data and Knowledge Integration for Health (CIDACS), Fundação Oswaldo Cruz, Salvador, Brazil;

2 Faculty of Epidemiology and Population Health, London School of Hygiene and Tropical Medicine, London, UK;

3 The Gonçalo Moniz Institute (IGM), Fundação Oswaldo Cruz, Salvador, Brazil.

4 Faculty of Medicine of Bahia, Universidade Federal da Bahia, Salvador, Brazil.read

5 Institute of Collective Health (ISC), Federal University of Bahia, Brazil

Contents

Supplementary Material 1: The details of each dataset and variables obtained from the dataset, as well as the information on the linkage. 3

Material 1.1: Databases used in the analysis, obtained from the cohort profile..3

Material 1.3: Details on the linkage accuracy..5

Material 1.4: Details on SINAN registries excluded based on final confirmation status (modification during the revision process). 5

Supplementary Table 1: The characteristics of the matched and the non-matched controls, as well as the P value and SMD for the comparison of the exposed and the matched non-exposed. 8

Supplementary Table 2 The number of events overall and in each stratum..10

Supplementary Table 3: The number of neonatal events in each stratum. 10

Supplementary Table 4: HR of the Cox regressions and their 95% CI obtained by bootstrapping..11

Supplementary Table 5: Standardized marginal risk and absolute risk difference per 1000 population for each exposure group, and their 95% confidence interval (CI) obtained by bootstrapping 11

Supplementary Table 6: Proportional hazards assumption test by Schoenfeld residuals. 12

Supplementary Table 7: HR of the Cox regressions of neonatal outcomes and their 95% CI obtained by bootstrapping..13

Supplementary Table 8: The ICD-10 codes and classes for the causes of the first-time hospitalizations..13

Supplementary Table 9 : The number of observations and events in each sensitivity analyses. 14

Supplementary Table 10: The HR for the Cox regression of the sensitivity analyses..15

Supplementary Table 11: The distribution of missing variables among observations with at least one variable missing, and those with complete variables. 15

Supplementary Table 12: STORBE checklist. 17

Supplementary Figure 1: The distribution of causes of first-time hospitalization by CHIKF exposure status. ..20

Supplementary Figure 2: DAG model for the covariates included in the study. 21

Reference 21

Supplementary Material 1: The details of each dataset and variables obtained from the dataset, as well as the information on the linkage.

Material 1.1: Databases used in the analysis, obtained from the cohort profile

Information cited from the cohort profile described in detail in Paixão 2024(1)

Database	Description	Variable used	Sensitivity
SINASC (Sistema de Informação sobre Nascidos Vivos/ Information System of Live Birth)	SINASC is the birth registry of Brazil, completed by a health professional who was present at the delivery	Information about the mother (age, educational attainment, marital status, and race/ethnicity), the pregnancy (the length of gestation, the number of previous gestations, whether the pregnancy was a singleton, and the number of antenatal care visits), and the newborn (birth date, sex, the municipality of residence, birth weight, gestational age at birth, and the APGAR score at 1 minute and 5 minutes).	Records information of 97% of Brazilian live births
CadUnico (Cadastro único para Programas Sociais/ Unified System for Social Programs	CadUnico is an integrated system in which all households and individuals receiving any social programs, including the conditional cash transfer program Bolsa Familia, are registered. It contains socioeconomic information about these families.	Whether the mother of the newborn is registered in the CadUnico, and the urbanicity of the residence.	More than 50% of the Brazilian population
SINAN (Sistema de Informação sobre Agravos de Notificação/ Information System for Notifiable Diseases)	SINAN contains all the registered cases of specific infectious diseases including Chikungunya, Dengue and Zika. The health professional who diagnosed these infections in the local health center registers all cases in this system. The system is disease specific.	Date of symptom onset, date of registry, and the diagnosis (either clinical or lab-confirmed) of the CHIKV, DENV and ZIKV infection	Depends on the disease

SIM (Sistema de Informação sobre Mortalidade/ Information System of Mortality)	SIM uses the death certificate, a legal document that records all deaths.	Date of death	Varies by place, range 70–95% of Brazilian deaths
SIH (Sistema de Informações Hospitalares/ Hospitalization Information System)	SIH is the register for all hospital admissions in the public hospital, under the national health system (Sistema Único de Saude). Admissions in private hospitals are not included, but it is probable that the poorer population registered in CadUnico uses the public hospitals.	Date of hospitalization and the cause (ICD-10) of hospitalization	Around 70% of all hospitalizations in Brazil

Material 1.2: Details on the exclusion criteria

Criteria	Numbers	Reason
Births with mismatched birth dates between SINASC and SIH	21064	Remove data with inconsistencies in the chronology of events, which has a high probability of linkage error
Does not have an RGI	235	The RGI was used for matching; thus, the information was needed for selecting the cohort.
Non-singleton births	123922	Having multiple fetuses is a high risk of birth complications and neonatal morbidity, thus the outcome may be caused by this rather than the infection.
Births with inconsistent dates (date for hospitalization or death before the date of birth)	119	Remove data with inconsistencies in the chronology of events, which has a high probability of linkage error
Births with missing gestational age at birth	130969	Gestational age information is needed to define the timing of exposure.
Births from mothers younger than 14.9 years or older than 49.9 Years	56657	The reproductive age defined by the WHO is 15 to 49 years. There is high possibility that the extreme values were errors in the data record, and if the value is true, the young (or old) maternal age is associated with birth complications.
Births with weights of below 500g or above 6000g	7040	There is high possibility of error in data record, and even if the value is true, that the newborn was hospitalized or died due to the underlying condition that caused a low birth weight.

Births with gestational ages below 20 weeks or above 45 weeks	479	There is high possibility of error in data record, and even if the value is true, that the newborn was hospitalized or died due to prematurity rather than the infection
Births exposed to in-utero DENV or ZIKV	41136	The outcome of event (hospitalization or death) may have been associated with the exposure to other Arboviruses, especially given that ZIKV is known to cause congenital Zika syndrome
Births with inconsistent CHIKV infection status (more than 2 infection registries which were more than 30 days apart)	11	It was uncertain whether both registries were of the same episode, and there was high possibility that at least one of the registries was a misclassification. Furthermore, we were not sure which of the multiple registries was the actual date the symptom started.
Births with CHIKV symptom onset before January 1st 2015	19	Although there were some CHIKV cases reported in specific regions during 2014, the disease diagnosis protocol had not been well established before the outbreak in 2015, thus there was a high possibility of misclassification.

Material 1.3: Details on the linkage accuracy

A detailed information is available in Ali 2019(2) and Paixao 2024(1). In summary, record linkage was performed using CIDACS-RL, a validated tool developed for large-scale Brazilian administrative data. This approach combines deterministic and probabilistic matching techniques, utilizing key identifiers including name, mother's name, date of birth, sex, and municipality of residence. Prior to linkage, datasets underwent rigorous preprocessing, including standardization of names and dates, imputation of missing values, and removal of duplicates.

The CIDACS-RL algorithm uses Apache Lucene for indexing and blocking to reduce computational demands, followed by a hybrid matching approach. Categorical variables were matched deterministically, while names and dates were compared probabilistically using similarity functions like the Dice coefficient. The linkage process was conducted within the secure data infrastructure of CIDACS, adhering to strict data security protocols and ethical governance. CIDACS-RL has been previously validated with >90% sensitivity and specificity.

Material 1.4: Details on SINAN registries excluded based on final confirmation status (modification during the revision process).

After our initial submission to *Nature Communications* using 3,154 exposed cases and 31,540 matched controls, we refined the exposed population to 1,821 cases in the revised analyses. Initially, we defined exposure as any individual registered in SINAN CHIK registry. However, SINAN notifications represent an initial clinical suspicion at the time of care and are subsequently followed by a standardized epidemiological investigation.

In Brazil, all suspected CHIK cases notified to SINAN undergo an epidemiological investigation, conducted by local or municipal surveillance teams under the coordination of the Ministry of Health (3). This investigation typically occurs within days to weeks after notification and includes review of clinical evolution, epidemiological context (such as local transmission and travel history), and laboratory test results when available. Based on this investigation, each notification is reclassified into one of the following mutually exclusive categories: confirmed, discarded, or inconclusive.

Classification by the epidemiological investigation	N
Confirmed CHIK	1821
Denied	651
Inconclusive	328
Missing	362

In the present revision, we restricted the exposed group to confirmed CHIK cases only, excluding notifications that were subsequently classified as discarded or inconclusive. This decision was made to improve exposure specificity and to minimize non-differential exposure misclassification arising from inclusion of suspected cases that were later ruled out or could not be definitively classified.

To assess whether this refinement materially altered the profile of the exposed population, we compared baseline maternal and child characteristics between the newly defined exposed group (N = 1,821) and the exposed population in the previous definition (N = 3,154). The baseline characteristics were highly comparable between groups, indicating that restriction to confirmed cases did not meaningfully change the underlying population structure.

	All observations registered in SINAN as CHIK-suspected (N=3,154)	CHIK cases confirmed after epidemiological investigation (N=1,821)
**Sex**		
Female	1522 (48.3)	892 (49.0)
Male	1631 (51.7)	928 (51.0)
missing	1 (0.0)	1 (0.1)
**Birth weight (grams)**		
mean (SD)	3255.91 (550.91)	3255.45 (540.35)
501-1000g	11 (0.3)	6 (0.3)
1001-1500g	17 (0.5)	11 (0.6)
1501-2500g	198 (6.3)	102 (5.6)
2501-4000g	2714 (86.0)	1584 (87.0)
4001-6000g	214 (6.8)	118 (6.5)
missing	0 (0.0)	0 (0.0)
**Gestational age at birth, weeks**		
mean (SD)	38.73 (2.20)	38.83 (2.14)
20-27	14 (0.4)	7 (0.4)
28-31	26 (0.8)	13 (0.7)
32-36	254 (8.1)	139 (7.6)
37-46	2860 (90.7)	1662 (91.3)
**Marital status**		
Divorced	25 (0.8)	12 (0.7)
Married	625 (19.8)	360 (19.8)
Single	1496 (47.4)	900 (49.4)

Stable union	977 (31.0)	531 (29.2)
Widowed	9 (0.3)	5 (0.3)
missing	22 (0.7)	13 (0.7)
**Maternal education, years**		
No education	17 (0.5)	10 (0.5)
1-3 years	118 (3.7)	71 (3.9)
4-7 years	693 (22.0)	390 (21.4)
8-11 years	2103 (66.7)	1244 (68.3)
12 or more years	223 (7.1)	106 (5.8)
**Maternal race/ethnicity**		
Asian	8 (0.3)	2 (0.1)
Black (Preto)	112 (3.6)	68 (3.7)
Indigenous	9 (0.3)	6 (0.3)
Mixed (Pardo)	2693 (85.4)	1571 (86.3)
White	332 (10.5)	174 (9.6)
**Maternal age, years**		
mean (SD)	25.98 (6.51)	25.90 (6.56)
15 to 19	587 (18.6)	345 (18.9)
20 to 34	2227 (70.6)	1283 (70.5)
35 to 49	340 (10.8)	193 (10.6)
**Number of ANC compared to recommended for gestational age**		
Adequate	2611 (82.8)	1511 (83.0)
One time less	251 (8.0)	133 (7.3)
Two or more times less	292 (9.3)	177 (9.7)
**Previous pregnancies**		
None	1088 (34.5)	618 (33.9)
One or more	2066 (65.5)	1203 (66.1)
**Urbanicity of residence**		
Rural	651 (20.6)	310 (17.0)
Urban	2503 (79.4)	1511 (83.0)
**Year of conception**		
2014	83 (2.6)	18 (1.0)
2015	1322 (41.9)	654 (35.9)
2016	997 (31.6)	613 (33.7)
2017	664 (21.1)	472 (25.9)
2018	88 (2.8)	64 (3.5)
**All-cause hospitalization up to age 3**		
No	2467 (78.2)	1428 (78.4)
Yes	686 (21.8)	393 (21.6)

**All-cause death up to age 3**		
No	3116 (98.8)	1800 (98.8)
Yes	38 (1.2)	21 (1.2)

Supplementary Table 1: The characteristics of the matched and the non-matched controls, as well as the P value and SMD for the comparison of the exposed and the matched non-exposed.

	Not matched (all non-exposed, N=4,641,159)	Matched				
		Overall N = 34,694	Non-exposed N = 31,540	Exposed N = 3,154	P value	SMD
**Sex**						
Female	2262709 (48.8)	9741 (48.6)	8849 (48.6)	892 (49.0)	0.325	0.026
Male	2377742 (51.2)	10287 (51.4)	9359 (51.4)	928 (51.0)		
Unknown	708 (0.0)	3 (0.0)	2 (0.0)	1 (0.1)		
**Birth weight category**						
mean (SD)	3217.01 (531.95)	3249.46 (534.66)	3248.86 (534.10)	3255.45 (540.35)	0.616	0.012
501-1000g	17517 (0.4)	77 (0.4)	71 (0.4)	6 (0.3)	0.968	0.026
1001-1500g	27694 (0.6)	109 (0.5)	98 (0.5)	11 (0.6)		
1501-2500g	297149 (6.4)	1160 (5.8)	1058 (5.8)	102 (5.6)		
2501-4000g	4052386 (87.3)	17447 (87.1)	15863 (87.1)	1584 (87.0)		
4001-6000g	245113 (5.3)	1236 (6.2)	1118 (6.1)	118 (6.5)		
Unknown	1300 (0.0)	2 (0.0)	2 (0.0)	0 (0.0)		
**Gestation week category**						
mean (SD)	38.65 (2.14)	38.72 (2.22)	38.71 (2.23)	38.83 (2.14)	0.024	0.056
20-27wks	18411 (0.4)	89 (0.4)	82 (0.5)	7 (0.4)	0.134	0.06
28-31wks	41640 (0.9)	180 (0.9)	167 (0.9)	13 (0.7)		
32-36wks	413990 (8.9)	1800 (9.0)	1661 (9.1)	139 (7.6)		
37-46wks	4167118 (89.8)	17962 (89.7)	16300 (89.5)	1662 (91.3)		
**Marital status**						
Divorced	48447 (1.0)	137 (0.7)	125 (0.7)	12 (0.7)	0.344	0.058
Married	1007037 (21.7)	4313 (21.5)	3953 (21.7)	360 (19.8)		
Single	2324849 (50.1)	9499 (47.4)	8599 (47.2)	900 (49.4)		
Stable union	1228730 (26.5)	5916 (29.5)	5385 (29.6)	531 (29.2)		
Widowed	7328 (0.2)	38 (0.2)	33 (0.2)	5 (0.3)		
missing	24768 (0.5)	128 (0.6)	115 (0.6)	13 (0.7)		

**Maternal education, years**						
No education	22361 (0.5)	126 (0.6)	116 (0.6)	10 (0.5)		
1-3 years	130268 (2.8)	744 (3.7)	673 (3.7)	71 (3.9)	0.003	0.101
4-7 years	995010 (21.4)	4666 (23.3)	4276 (23.5)	390 (21.4)		
8-11 years	3132721 (67.5)	12969 (64.7)	11725 (64.4)	1244 (68.3)		
12 or more years	360799 (7.8)	1526 (7.6)	1420 (7.8)	106 (5.8)		
**Maternal race/ethnicity**						
Asian	13338 (0.3)	42 (0.2)	40 (0.2)	2 (0.1)	0.059	0.078
Black	297458 (6.4)	867 (4.3)	799 (4.4)	68 (3.7)		
Indigenous	47879 (1.0)	114 (0.6)	108 (0.6)	6 (0.3)		
Mixed (Pardo)	2970071 (64.0)	16827 (84.0)	15256 (83.8)	1571 (86.3)		
White	1312413 (28.3)	2181 (10.9)	2007 (11.0)	174 (9.6)		
**Maternal age category**						
Mean (SD)	25.37 (6.40)	25.42 (6.43)	25.38 (6.42)	25.90 (6.56)	0.001	0.081
15 to 19	960332 (20.7)	4092 (20.4)	3747 (20.6)	345 (18.9)	0.257	0.041
20 to 34	3210853 (69.2)	13866 (69.2)	12583 (69.1)	1283 (70.5)		
35 to 49	469974 (10.1)	2073 (10.3)	1880 (10.3)	193 (10.6)		
**Number of antenatal care visits**						
Adequate	3636937 (78.4)	15648 (78.1)	14137 (77.6)	1511 (83.0)		
One time less	390761 (8.4)	1811 (9.0)	1678 (9.2)	133 (7.3)		
Two or more times less	613461 (13.2)	2572 (12.8)	2395 (13.2)	177 (9.7)	<0.001	0.136
**Number of previous gestations**						
None	1622366 (35.0)	7002 (35.0)	6384 (35.1)	618 (33.9)	0.352	0.024
One or more	3018793 (65.0)	13029 (65.0)	11826 (64.9)	1203 (66.1)		
**Urbanicity of residence**						
Rural	1036885 (22.3)	5313 (26.5)	5003 (27.5)	310 (17.0)	<0.001	0.253
Urban	3604274 (77.7)	14718 (73.5)	13207 (72.5)	1511 (83.0)		
**Ever hospitalized**	911486 (19.6)	3635 (18.1)	3242 (17.8)	393 (21.6)	<0.001	0.095
**Death**	48378 (1.0)	215 (1.1)	194 (1.1)	21 (1.2)	0.820	0.008

Supplementary Table 2 The number of events overall and in each stratum. Error! Bookmark not defined.

	Total number	Admissions		Death	
		Number	Percentage	Number	Percentage
Overall	20031	3735	18.65%	216	1.08%
Unexposed	18210	3242	17.8%	195	1.07%
Exposed	1821	393	21.58%	21	1.15%
First	536	118	22.01%	5	0.93%
Second	713	161	22.58%	11	1.54%
Third	572	114	19.93%	5	0.87%
Antepartum	1761	373	21.18%	17	0.97%
Intrapartum	60	20	33.33%	4	6.67%

Supplementary Table 3: The number of neonatal events in each stratum.

	Total number	Admissions		Death	
		Number	Percentage	Number	Percentage
Overall	20031	2030	10.13%	131	0.65%
Unexposed	18210	1825	10.02%	117	0.64%
Exposed	1821	205	11.26%	14	0.77%
First	536	60	11.19%	2	0.37%
Second	713	88	12.34%	8	1.12%
Third	572	57	9.97%	4	0.7%
Antepartum	1761	193	10.96%	11	0.62%
Intrapartum	60	12	20%	3	5%

Supplementary Table 4: HR of the Cox regressions and their 95% CI obtained by bootstrapping

		HR	Bootstrapped 95% CI
Admission	Overall	1.213	[1.105-1.361]
	First trimester	1.349	[1.100-1.667]
	Second trimester	1.25	[1.055-1.479]
	Third trimester	1.057	[0.862-1.299]
	Antepartum	1.187	[1.065-1.348]
	Intrapartum	2.079	[1.330-3.442]
Death	Overall	1.014	[0.575-1.617]
	First trimester	0.687	[0.141-1.605]
	Second trimester	1.458	[0.671-2.985]
	Third trimester	0.842	[0.164-1.748]
	Antepartum	0.843	[0.482-1.292]
	Intrapartum	6.169	[0.942-34.90]

Supplementary Table 5: Standardized marginal risk and absolute risk difference per 1000 population for each exposure group, and their 95% confidence interval (CI) obtained by bootstrapping

		12 months			24 months			36 months		
		Risk per 1000 people	Risk difference per 1000 people	95% CI	Risk per 1000 people	Risk difference per 1000 people	95% CI	Risk per 1000 people	Risk difference per 1000 people	95% CI
Admission	Non-exposed	185.9			185.9			255.6		
	CHIK	217.3	31.4	[18.3-47.6]	265	36.7	[21.6-55.3]	295	39.4	[23.3-59.2]
Death	non-exposed	17.9			19.6			20.3		
	CHIK	19.1	1.2	[-4.2-8.4]	20.9	1.3	[-4.5-9]	21.6	1.3	[-4.7-9.2]

Supplementary Table 6: Proportional hazards assumption test by Schoenfeld residuals.

Cox model	Exposure variable	p value	PH Assumption Violated
**Main analysis for admissions**			
Overall	CHIK	0.068	No
Analyses by trimester	trimester	0.056	No
Analyses by intrapartum period	intrapartum	0.166	No
**Main analysis for deaths**			
Overall	CHIK	0.245	No
Analyses by trimester	trimester	0.674	No
Analyses by intrapartum period	intrapartum	0.258	No
**Sub-analyses for neonatal outcomes**			
Admissions	CHIK	0.57	No
Deaths	CHIK	0.03	Yes
**Sensitivity analyses for normal-weight and -term births**			
Admissions	CHIK	0.257	No
Deaths	CHIK	0.066	No
**Sensitivity analyses for lab-confirmed cases**			
Admissions	CHIK	0.008	Yes
Deaths	CHIK	0.916	No
**Sensitivity analyses using La Reunionese definition of exposure period (including exposures until 2 days after birth)**			
Admissions, overall	CHIK	0.062	No
Admissions, intrapartum period	intrapartum	0.128	No
Death, overall	CHIK	0.556	No
Death, intrapartum period	intrapartum	0.31	No
**Sensitivity analyses for males**			
Admissions	CHIK	0.418	No
**Sensitivity analyses for females**			
Admissions	CHIK	0.359	No

Supplementary Table 7: HR of the Cox regressions of neonatal outcomes and their 95% CI obtained by bootstrapping.

	HR	Bootstrapped 95% CI
Neonatal Admission	1.074	[0.928-1.262]
Neonatal Death	1.115	[0.489-1.946]

Supplementary Table 8: The ICD-10 codes and classes for the causes of the first-time hospitalizations.

Letter(s)	ICD Chapter Title	Non exposed (N=18,210)		Exposed (N=1.821)	
		N	%	N	%
A–B	Certain infectious and parasitic diseases	524	16.2	62	15.8
C–D	Neoplasms and Diseases of the blood and blood-forming organs and certain disorders involving the immune mechanism	27	0.8	2	0.5
E	Endocrine, nutritional and metabolic diseases	27	0.8	3	0.8
F	Mental and behavioral disorders	0	0	0	0
G	Diseases of the nervous system	40	1.2	3	0.8
H	Diseases of the eye, adnexa, ear and mastoid process	11	0.3	3	0.8
I	Diseases of the circulatory system	9	0.3	2	0.5
J	Diseases of the respiratory system	702	21.7	78	19.8
K	Diseases of the digestive system	53	1.6	11	2.8
L	Diseases of the skin and subcutaneous tissue	71	2.2	8	2
M	Diseases of the musculoskeletal system and connective tissue	5	0.2	1	0.3
N	Diseases of the genitourinary system	79	2.4	16	4.1
O	Pregnancy, childbirth and the puerperium	2	0.1	0	0
P	Certain conditions originating in the perinatal period	1480	45.7	174	44.3
Q	Congenital malformations, deformations and chromosomal abnormalities	92	2.8	13	3.3
R	Symptoms, signs and abnormal clinical and laboratory findings, not elsewhere classified	32	1	3	0.8
S–T	Injury, poisoning and certain other consequences of external causes	49	1.5	6	1.5
V–Y	External causes of morbidity and mortality	0	0	0	0

Z	Factors influencing health status and contact with health services	39	1.2	8	2

Supplementary Table 9 : The number of observations and events in each sensitivity analyses.

	Total number	Admissions		Death	
		Number	Percentage	Number	Percentage
**For normal-weight, normal-term births**					
Non-exposed	13934	2150	15.4%	70	0.5%
Exposed	1610	302	18.8%	7	0.4%
**For laboratory-confirmed exposed cases**					
Non-exposed	6990	1254	17.9%	64	0.9%
Exposed	699	124	17.7%	6	0.9%
**Exposure definition by La Reunionese team (conception to 2 days after birth)**					
Non-exposed	18500	3327	18%	199	1.1%
Antepartum	1809	387	21.4%	21	1.2%
Intrapartum	29	13	44.8%	2	6.9%
**Stratified by sex**					
**Male**					
Non-exposed	4746	862	18.2%	50	1.1%
Exposed	928	219	23.6%	6	0.6%
**Female**					
Non-exposed	4310	728	16.9%	50	1.2%
Exposed	892	174	19.5%	15	1.7%

Supplementary Table 10: The HR for the Cox regression of the sensitivity analyses.

	HR	Bootstrapped 95% CI
**For normal-weight, normal-term births**		
Admission	1.24	1.09-1.40
Death	0.8	0.36-1.75
**For laboratory-confirmed exposed cases**		
Admission	1.02	0.85-1.23
Death	0.96	0.41-1.33
**Exposure definition by La Reunionese team**		
Admission		
Overall	1.22	1.09-1.36
Intrapartum	4.25	2.14-8.44
Death		
Overall	1.07	0.68-1.69
Intrapartum	2.72	0.45-16.48
**Sext stratified**		
**Male**		
Admission	1.32	1.12-1.56
**Female**		
Admission	1.21	1.01-1.46

Supplementary Table 11: The distribution of missing variables among observations with at least one variable missing, and those with complete variables.

	**Observations with missing covariates**		**Observations without missing covariates**	
	Overall N = 750,793	Exposed N =497	Overall N =4,661,190	Exposed N = 1,821
**Sex**				
Female	366717 (48.8)	228 (45.9)	2272450 (48.8)	892 (49.0)
Male	383852 (51.1)	269 (54.1)	2388029 (51.2)	928 (51.0)
NA	224 (0.0)	0 (0.0)	711 (0.0)	1 (0.1)
**Birth weight**				
mean (SD)	3202.32 (548.74)	3199.90 (573.85)	3217.15 (531.97)	3255.45 (540.35)
501-1000g	3810 (0.5)	5 (1.0)	17594 (0.4)	6 (0.3)
1001-1500g	5306 (0.7)	4 (0.8)	27803 (0.6)	11 (0.6)
1501-2500g	52073 (6.9)	32 (6.4)	298309 (6.4)	102 (5.6)

2501-4000g	650169 (86.6)	433 (87.1)	4069833 (87.3)	1584 (87.0)
4001-6000g	39045 (5.2)	23 (4.6)	246349 (5.3)	118 (6.5)
NA	390 (0.1)	0 (0.0)	1302 (0.0)	0 (0.0)
**Gestational weeks**				
mean (SD)	38.72 (2.33)	38.61 (2.25)	38.65 (2.14)	38.83 (2.14)
20-27wks	4235 (0.6)	1 (0.2)	18500 (0.4)	7 (0.4)
28-31wks	8200 (1.1)	10 (2.0)	41820 (0.9)	13 (0.7)
32-36wks	69130 (9.2)	47 (9.5)	415790 (8.9)	139 (7.6)
37-46wks	669228 (89.1)	439 (88.3)	4185080 (89.8)	1662 (91.3)
**Marital status**				
Divorced	5711 (0.8)	3 (0.6)	48584 (1.0)	12 (0.7)
Married	160930 (21.4)	116 (23.3)	1011350 (21.7)	360 (19.8)
Single	342844 (45.7)	202 (40.6)	2334348 (50.1)	900 (49.4)
Stable union	206089 (27.4)	153 (30.8)	1234646 (26.5)	531 (29.2)
Widowed	1238 (0.2)	3 (0.6)	7366 (0.2)	5 (0.3)
NA	33981 (4.5)	20 (4.0)	24896 (0.5)	13 (0.7)
**Maternal education**				
none	8337 (1.1)	4 (0.8)	22487 (0.5)	10 (0.5)
1-3 years	30501 (4.1)	13 (2.6)	131012 (2.8)	71 (3.9)
4-7 years	152699 (20.3)	91 (18.3)	999676 (21.4)	390 (21.4)
8-11 years	420221 (56.0)	301 (60.6)	3145690 (67.5)	1244 (68.3)
12 or more years	63322 (8.4)	36 (7.2)	362325 (7.8)	106 (5.8)
NA	75713 (10.1)	52 (10.5)		
**Maternal race**				
Asian	1446 (0.2)	0 (0.0)	13380 (0.3)	2 (0.1)
Black	31171 (4.2)	2 (0.4)	298325 (6.4)	68 (3.7)
Indigenous	7625 (1.0)	0 (0.0)	47993 (1.0)	6 (0.3)
Brown	368455 (49.1)	233 (46.9)	2986898 (64.1)	1571 (86.3)
White	106426 (14.2)	23 (4.6)	1314594 (28.2)	174 (9.6)
NA	235670 (31.4)	239 (48.1)		
**Maternal age**				
mean (SD)	25.31 (6.37)	26.26 (6.61)	25.37 (6.40)	25.90 (6.56)
15 to 19	149943 (20.0)	84 (16.9)	964424 (20.7)	345 (18.9)
20 to 34	526272 (70.1)	348 (70.0)	3224719 (69.2)	1283 (70.5)
35 to 49	74567 (9.9)	65 (13.1)	472047 (10.1)	193 (10.6)
NA	11 (0.0)	0 (0.0)		
**Urbanicity**				
Rural	172540 (23.0)	59 (11.9)	1042198 (22.4)	310 (17.0)
Urban	419592 (55.9)	356 (71.6)	3618992 (77.6)	1511 (83.0)

NA	158661 (21.1)	82 (16.5)		
**All-cause hospitalization up to age 3**				
No	599523 (79.9)	343 (69.0)	3746018 (80.4)	1428 (78.4)
Yes	151270 (20.1)	154 (31.0)	915172 (19.6)	393 (21.6)
**All-cause death up to age 3**				
No	740998 (98.7)	489 (98.4)	4612596 (99.0)	1800 (98.8)
Yes	9795 (1.3)	8 (1.6)	48594 (1.0)	21 (1.2)

Supplementary Table 12: STORBE checklist.

	Item No	Recommendation
**Title and abstract**	1	(*a*)The study design, longitudinal study, is stated in the title
		(*b*) The methods section of the abstract summarizes what we did, and the results section summarizes what was found.
**Introduction**		
Background/rationale	2	The scientific background and rationale for the investigation is explained in the second and third paragraph of the introduction
Objectives	3	The objectives of ths study is stated in the last paragraph of the introduction
**Methods**		
Study design	4	The key elements of the study design (a matched cohort study following children from birth until hospitalization, death, age 3, or Dec 31, 2018) are described at the beginning of the Methods, under Study design
Setting	5	The study setting, population, and time frame are described under Study design and Study population. Children were born between Jan 1, 2015 and Dec 31, 2018 in Brazil. Data were obtained from linked administrative databases (CIDACS Birth Cohort, CADU, SINASC, SINAN, SIH, SIM). Follow-up extended to Dec 31, 2018, with periods of exposure and outcomes clearly defined.
Participants	6	(*a*) Eligibility criteria, sources, and selection are described under Study population. Exclusions included non-singletons, implausible dates, missing gestational age, implausible birthweight, and exposure to ZIKV or DENV. Participants were followed until hospitalization, death, age 3, or end of 2018.
		(*b*) Matching criteria are given under Statistical analysis: each exposed newborn was matched to 10 unexposed by month of conception and Immediate Geographic Region (RGI). Final numbers were 3,154 exposed and 31,540 unexposed.

Variables	7	Definitions of exposures, outcomes, predictors, confounders, and effect modifiers are described in Exposures, Outcome, and Covariates. Exposures: maternal CHIKF infection during pregnancy, stratified by trimester and intrapartum. Outcomes: first hospitalization and all-cause death by age 3. Covariates included maternal education, race/skin colour, age, adequacy of antenatal care, previous gestations, and urbanicity.
Data sources/ measurement	8	Sources of data and methods of assessment are detailed under Data source. Maternal and child data came from SINASC, social information from CADU, exposures from SINAN, hospitalizations from SIH, and deaths from SIM. Assessment methods were consistent across exposed and unexposed groups because they rely on the same national surveillance systems.
Bias	9	Efforts to reduce bias are described in Statistical analysis and Limitations. Matching by conception month and RGI reduced temporal and geographic confounding. Excluding records with implausible or inconsistent data minimized misclassification. Sensitivity analyses (lab-confirmed cases, alternative exposure definitions, restriction to term/normal weight births) addressed possible misclassification and residual confounding
Study size	10	Study size was determined by including all eligible live births in the CIDACS Birth Cohort between 2015 and 2018 after applying exclusion criteria. No formal sample size calculation was performed.
Quantitative variables	11	Quantitative variables were categorized where appropriate (e.g., maternal age into 3 groups, gestational age into term/preterm, education into categories). Some categories were collapsed (e.g., maternal education none/1–3 years, race white/non-white) to ensure sufficient statistical power
Statistical methods	12	(*a*) Statistical methods are described under Statistical analysis: stratified Cox proportional hazards models adjusted for confounders estimated HRs, with marginal standardized risk differences. Kaplan–Meier curves described survival. Bootstrapping was used for confidence intervals.
		(*b*) Subgroup analyses included trimester-specific and intrapartum exposures; interactions were assessed by categorizing exposure timing.
		(*c*) Missing data were minimal (<6%); a complete case analysis was performed.
		(*d*) All hospitalizations and deaths are registered in the national registry, thus there were no loss to follow-up
		(*e*) Four sensitivity analyses were conducted: restriction to term/normal weight births, restriction to lab-confirmed cases, extended exposure window to two days post-birth, and alternative matching criteria (4:1 matching).
**Results**		
Participants	13*	(a) Numbers of individuals at each stage are reported in Results, Study population and baseline characteristics and shown in Figure 1. From 5,795,265 live births, exclusions were applied, leaving 3,154 exposed and 31,540 matched unexposed.

		(b) Reasons for non-participation (exclusions: non-singletons, implausible dates, implausible birthweight, missing gestational age, other arbovirus exposures, inconsistent CHIKF records) are described in Study population (Methods).
		(c) A flow diagram of inclusion/exclusion is provided in Figure 1.
Descriptive data	14*	(a) Characteristics of study participants (demographics, maternal and pregnancy variables, covariates) are shown in Table 1 and Supplementary Table 4.
		(b) Numbers of missing data for each variable are given in Supplementary Table 2.
		(c) Follow-up time is summarized in Results: median follow-up for hospitalization was 19.1 months [IQR: 27.9], and for death was 25.8 months [IQR: 23.2].
Outcome data	15*	Numbers of outcome events are reported in Results. Hospitalizations: 687 (21%) in exposed and 5,705 (18%) in unexposed. Deaths: 38 (1.2%) in exposed and 342 (1.1%) in unexposed. Further details by cause are shown in Supplementary Tables 5–11 and Figures 2–4.
Main results	16	(*a*) The crude hazards are described as Kaplan Meier curves. Confounder-adjusted hazard ratios and risk differences with 95% CIs are reported in Results, All-cause first hospitalization and All-cause death, adjusted for maternal education, race/skin colour, age, antenatal care, previous gestations, and urbanicity.
		(*b*) Category boundaries are defined in Covariates (e.g., maternal age 15–19, 20–34, 35–49; education collapsed into categories; race white vs non-white).
		(*c*) Absolute risks and standardized risk differences per 1000 children are presented in Results, All-cause hospitalization (e.g., 39.4 additional hospitalizations per 1000 exposed).
Other analyses	17	Analyses of subgroups (by trimester, intrapartum, neonatal outcomes) and sensitivity analyses (term/normal weight births, lab-confirmed cases, extended exposure window, alternative matching) are described in Results, Sensitivity analyses and Methods, Statistical analysis.
**Discussion**		
Key results	18	Key results are summarized in the first paragraph of Discussion: a 22% higher hazard of hospitalization and possible increased risk of death, especially with intrapartum and early-trimester exposures.
Limitations	19	Limitations are discussed in Discussion, including live birth bias, passive surveillance and misclassification, missing data, lack of confounders (income, living conditions), under-ascertainment, and reduced power in sensitivity analyses.
Interpretation	20	Interpretation is provided throughout Discussion, linking findings to previous literature, hypothesized biological mechanisms, and public health implications, while acknowledging uncertainty and imprecision of mortality estimates.
Generalisability	21	Generalisability is discussed in Discussion: findings are relevant to Brazil and globally, given the expansion of endemic areas to Europe with climate change and globalization.
**Other information**		

Funding	22	Funding sources (Wellcome Trust, Royal Society) are reported in the Abstract and under Funding at the end of the manuscript.

Supplementary Figure 1: The distribution of causes of first-time hospitalization by CHIKF exposure status.

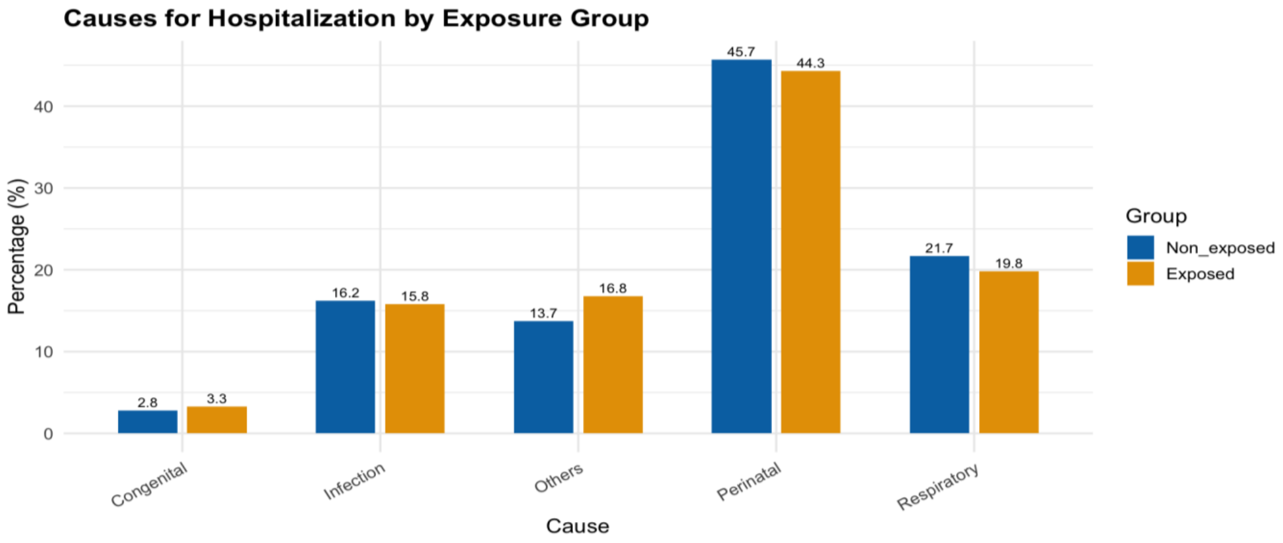

Supplementary Figure 2: DAG model for the covariates included in the study.

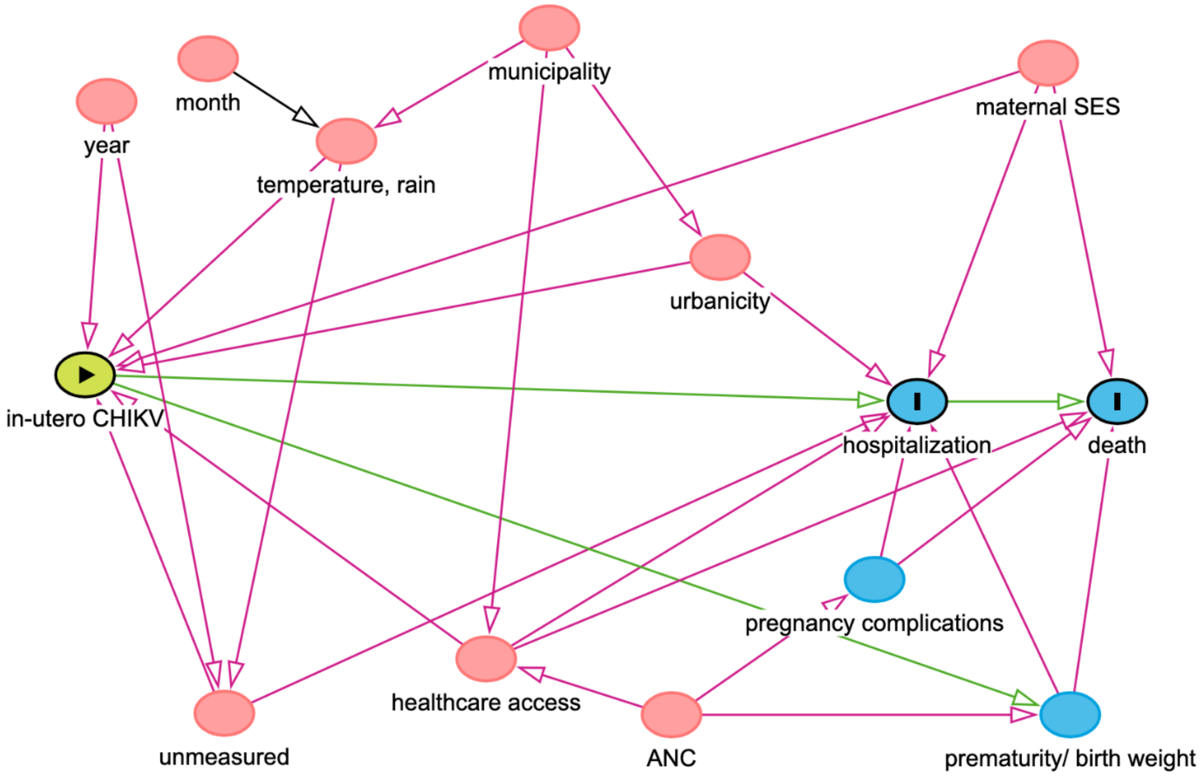

Abbreviations: ANC, antenatal care visits; CHIKV, chikungunya virus; SES, socioeconomic status

Reference

1. Paixao ES, Cerqueira-Silva T, Florentino PTV, Carroll O, Sanchez Clemente N, Lawlor DA, et al. A nationwide longitudinal investigation on the role of prenatal exposure to infectious diseases on the onset of chronic conditions in children and adolescents in Brazil. Wellcome Open Res. 2024 Oct 17;9:320.

2. Ali MS, Ichihara MY, Lopes LC, Barbosa GCG, Pita R, Carreiro RP, et al. Administrative data linkage in Brazil: Potentials for health technology assessment. Front Pharmacol. 2019 Sept 23;10:984.

3. Coelho GE, Martins J, Percio J, Santelli ACF e. S, Filippis AMB de, Brito CA, et al. Protocolo de investigação de óbitos por arbovírus urbanos no Brasil – dengue, chikungunya e zika [Internet]. Brasília: Ministério da Saúde; 2016. Available from: https://www.saude.ba.gov.br/wp-content/uploads/2022/11/Protocolo-Tecnico-INVESTIGACAO-OBITO-ARBOVIRUS.pdf
