# Supplementary material for: In-utero exposure to chikungunya and child morbimortality: a population-based study using linked routine data
Source: Nat Commun. 2026 Mar 21;17:4581. doi: 10.1038/s41467-026-70786-5 (PMC13194738; doi:10.1038/s41467-026-70786-5)
Supplement: Supplementary file 2 — Reporting Summary [file 41467_2026_70786_MOESM2_ESM.pdf]

nature portfolio

Corresponding author(s): Mio Kushibuchi

Last updated by author(s): Feb 21, 2026

Reporting Summary

Nature Portfolio wishes to improve the reproducibility of the work that we publish. This form provides structure for consistency and transparency in reporting. For further information on Nature Portfolio policies, see our Editorial Policies and the Editorial Policy Checklist.

Statistics

For all statistical analyses, confirm that the following items are present in the figure legend, table legend, main text, or Methods section.

n/a	Confirmed	
☐	☒	The exact sample size (*n*) for each experimental group/condition, given as a discrete number and unit of measurement
☐	☒	A statement on whether measurements were taken from distinct samples or whether the same sample was measured repeatedly
☐	☒	The statistical test(s) used AND whether they are one- or two-sided<br>*Only common tests should be described solely by name; describe more complex techniques in the Methods section.*
☐	☒	A description of all covariates tested
☐	☒	A description of any assumptions or corrections, such as tests of normality and adjustment for multiple comparisons
☐	☒	A full description of the statistical parameters including central tendency (e.g. means) or other basic estimates (e.g. regression coefficient) AND variation (e.g. standard deviation) or associated estimates of uncertainty (e.g. confidence intervals)
☐	☒	For null hypothesis testing, the test statistic (e.g. *F*, *t*, *r*) with confidence intervals, effect sizes, degrees of freedom and *P* value noted<br>*Give P values as exact values whenever suitable.*
☒	☐	For Bayesian analysis, information on the choice of priors and Markov chain Monte Carlo settings
☒	☐	For hierarchical and complex designs, identification of the appropriate level for tests and full reporting of outcomes
☒	☐	Estimates of effect sizes (e.g. Cohen's *d*, Pearson's *r*), indicating how they were calculated

Our web collection on statistics for biologists contains articles on many of the points above.

Software and code

Policy information about availability of computer code

Data collection	No custom code was developed for data collection, as we used routinely collected registry data.
Data analysis	All analyses were performed using R (version 4.2.1 and 4.4.1; R Core Team, R Foundation for Statistical Computing, Vienna, Austria). Matching was performed using the MatchIt package (version 4.5.5). Survival analyses, including Kaplan-Meier estimation, Cox proportional hazards modelling, Schoenfeld residual diagnostics, and bootstrapped confidence intervals, were conducted using the survival package (version 3.5.7) and survminer package (version 0.4.9). Standardised mean differences were calculated using the cobalt package (version 4.5.1). Data linkage, cleaning, and all data manipulation steps were performed using tidyverse packages (version 2.0.0), including dplyr and tidyr. Directed Acyclic Graph (DAG) construction was performed using DAGitty (version 3.0; http://www.dagitty.net). Figures were produced using ggplot2 (version 3.4.4) and survminer.

For manuscripts utilizing custom algorithms or software that are central to the research but not yet described in published literature, software must be made available to editors and reviewers. We strongly encourage code deposition in a community repository (e.g. GitHub). See the Nature Portfolio guidelines for submitting code & software for further information.

Data

Policy information about availability of data

All manuscripts must include a data availability statement. This statement should provide the following information, where applicable:

- Accession codes, unique identifiers, or web links for publicly available datasets
- A description of any restrictions on data availability
- For clinical datasets or third party data, please ensure that the statement adheres to our policy

The relevant data are included in the manuscript and appendix. Deidentified individual participant data are available on reasonable request to CIDACS (https://cidacs.bahia.fiocruz.br), subject to institutional collaboration agreements and Brazilian ethics committee approval.

Research involving human participants, their data, or biological material

Policy information about studies with human participants or human data. See also policy information about sex, gender (identity/presentation), and sexual orientation and race, ethnicity and racism.

Reporting on sex and gender	Biological sex reported by the healthcare provider at birth and registered in the birth registry SINASC was used to obtain the Cox models stratified by sex. Self-reported gender information was not obtainable.
Reporting on race, ethnicity, or other socially relevant groupings	Self-declared race as recorded in the Brazilian governmental registry systems were used. Race was self-declared among Preto (Afro-descendents), Pardo (mixed race, usually referred as "brown"), Branco (Euro-descendents), Amarelo (Asians), and Indigeno (Indigenous population).
Population characteristics	Eligible individuals were all singleton live-born infants between January 1, 2015, and December 31, 2018, to women aged 15 to 49 years who were registered in CADU. Live-born was defined as a birth registered in SINASC with gestational weeks greater than 20 and a birth weight above 500g.
Recruitment	All live born individuals in Brazil born between 2015 and 2018 were eligible, for we used routinely collected registry data.
Ethics oversight	The study was approved by approved by the Research Ethics Committee (CEP) of the Gonçalo Moniz Research Center (CAAE: 71054923.8.0000.0040) and LSHTM Ethics (Ref: 29560).

Note that full information on the approval of the study protocol must also be provided in the manuscript.

Field-specific reporting

Please select the one below that is the best fit for your research. If you are not sure, read the appropriate sections before making your selection.

☒ Life sciences ☐ Behavioural & social sciences ☐ Ecological, evolutionary & environmental sciences

For a reference copy of the document with all sections, see nature.com/documents/nr-reporting-summary-flat.pdf

Life sciences study design

All studies must disclose on these points even when the disclosure is negative.

Sample size	A-priori sample size calculation was not performed, for we used nationwide registry data, and all live born individuals born in years 2015 to 2018 in Brazil were eligible.
Data exclusions	We excluded records with inconsistent dates (events occurring before the date of birth, or conflicting birth dates across registries); births with missing gestational age; births with weights above 6000g; births with documented in utero exposure to ZIKV or DENV; and births with inconsistent CHIKF infection data (details given in the exposure section).
Replication	The developed code was independently checked by the First author and the second author to ensure accuracy and replication.
Randomization	This was an observational study and not applicable.
Blinding	This was an observational study and not applicable

Reporting for specific materials, systems and methods

We require information from authors about some types of materials, experimental systems and methods used in many studies. Here, indicate whether each material, system or method listed is relevant to your study. If you are not sure if a list item applies to your research, read the appropriate section before selecting a response.

Materials & experimental systems

n/a	Involved in the study
☒	☐ Antibodies
☒	☐ Eukaryotic cell lines
☒	☐ Palaeontology and archaeology
☒	☐ Animals and other organisms
☒	☐ Clinical data
☒	☐ Dual use research of concern
☒	☐ Plants

Methods

n/a	Involved in the study
☒	☐ ChIP-seq
☒	☐ Flow cytometry
☒	☐ MRI-based neuroimaging

Plants

Seed stocks

Report on the source of all seed stocks or other plant material used. If applicable, state the seed stock centre and catalogue number. If plant specimens were collected from the field, describe the collection location, date and sampling procedures.

Novel plant genotypes

Describe the methods by which all novel plant genotypes were produced. This includes those generated by transgenic approaches, gene editing, chemical/radiation-based mutagenesis and hybridization. For transgenic lines, describe the transformation method, the number of independent lines analyzed and the generation upon which experiments were performed. For gene-edited lines, describe the editor used, the endogenous sequence targeted for editing, the targeting guide RNA sequence (if applicable) and how the editor was applied.

Authentication

Describe any authentication procedures for each seed stock used or novel genotype generated. Describe any experiments used to assess the effect of a mutation and, where applicable, how potential secondary effects (e.g. second site T-DNA insertions, mosiacism, off-target gene editing) were examined.
